# Synchrotron Time-Lapse Imaging of Lignocellulosic Biomass Hydrolysis: Tracking Enzyme Localization by Protein Autofluorescence and Biochemical Modification of Cell Walls by Microfluidic Infrared Microspectroscopy

**DOI:** 10.3389/fpls.2018.00200

**Published:** 2018-02-20

**Authors:** Marie-Françoise Devaux, Frédéric Jamme, William André, Brigitte Bouchet, Camille Alvarado, Sylvie Durand, Paul Robert, Luc Saulnier, Estelle Bonnin, Fabienne Guillon

**Affiliations:** ^1^UR1268 Biopolymères Interactions et Assemblages, Institut National de la Recherche Agronomique Pays de la Loire, Nantes, France; ^2^Synchrotron SOLEIL, Gif-Sur-Yvette, France

**Keywords:** spectral imaging, maize stem cells, cellulose and hemicellulose degradation, lignin, image analysis

## Abstract

Tracking enzyme localization and following the local biochemical modification of the substrate should help explain the recalcitrance of lignocellulosic plant cell walls to enzymatic degradation. Time-lapse studies using conventional imaging require enzyme labeling and following the biochemical modifications of biopolymers found in plant cell walls, which cannot be easily achieved. In the present work, synchrotron facilities have been used to image the enzymatic degradation of lignocellulosic biomass without labeling the enzyme or the cell walls. Multichannel autofluorescence imaging of the protein and phenolic compounds after excitation at 275 nm highlighted the presence or absence of enzymes on cell walls and made it possible to track them during the reaction. Image analysis was used to quantify the fluorescence intensity variations. Consistent variations in the enzyme concentration were found locally for cell cavities and their surrounding cell walls. Microfluidic FT-IR microspectroscopy allowed for time-lapse tracking of local changes in the polysaccharides in cell walls during degradation. Hemicellulose degradation was found to occur prior to cellulose degradation using a Celluclast® preparation. Combining the fluorescence and FT-IR information yielded the conclusion that enzymes did not bind to lignified cell walls, which were consequently not degraded. Fluorescence multiscale imaging and FT-IR microspectroscopy showed an unexpected variability both in the initial biochemical composition and the degradation pattern, highlighting micro-domains in the cell wall of a given cell. Fluorescence intensity quantification showed that the enzymes were not evenly distributed, and their amount increased progressively on degradable cell walls. During degradation, adjacent cells were separated and the cell wall fragmented until complete degradation.

## Introduction

The development of plant biorefineries using lignocellulose biomass is a major challenge for the future in order to provide a substitute for using fossil carbon to produce biomolecules, bioenergy, and biomaterials (Maity, 2015). The biological conversion of biomass, involving microorganisms or derived enzymes, is attractive compared to the other main types of conversion used in industry because it offers the possibility of producing specific products in an environmentally friendly way. However, the bioconversion of biomass still remains economically challenging (Chundawat et al., 2011; Liguori and Faraco, 2016). A major current limitation is the incomplete deconstruction of lignocellulosic biopolymers. Lignocellulosic plants mainly consists of cell walls that may vary in composition depending on the plant species, tissues, or cell types. Several factors have been suggested to affect the bioconversion of lignocellulose (Anderson and Akin, 2008; Zhao et al., 2012; McCann and Carpita, 2015; Tan et al., 2016), with no consensus except for the detrimental role of lignin.

In recent papers, morphological and biochemical spatial heterogeneities were investigated to determine which factors limit the enzymatic degradation of plant cell walls. Real-time imaging has been used to study the action of cellulases and lytic polysaccharide monooxygenases on model celluloses during hydrolysis (Bubner et al., 2013; Luterbacher et al., 2013; Eibinger et al., 2014). These papers highlighted the morphological changes in the cellulose substrate using confocal laser scanning microscopy and high-speed atomic force microscopy. Though Raman or mid-infrared microspectroscopies are powerful techniques to investigate the spatial variability of polysaccharides and lignin (Muller and Polle, 2009; Allouche et al., 2012; Gierlinger, 2014; Largo-Gosens et al., 2014), only a few studies have reported the time-lapse studies of reactions. One challenge is acquiring the *in situ* spectra during reactions in a highly hydrated medium. Gierlinger et al. (2008) have used a custom-fluidic cell to follow the enzymatic degradation of cellulose in poplar wood sections. They showed that no changes were observed in lignified cell walls, while the gelatinous layer in tension wood completely disappeared. The time-lapse difference spectra of the degraded regions were quite similar to those of cellulose. In addition to imaging studies, Gillgren and Gorzsás (2016) adapted a set-up for the real-time tracking of a chemical reaction using FT-IR spectroscopy. They confirmed the potential of FT-IR time-lapse measurements to evaluate the reaction speed and the occurrence of intermediate species in the reaction in the context of lignocellulose.

Other papers have focused on the localization of enzymes during reactions. Several authors have used fluorescence confocal microscopy to map the localization of enzymes on lignocellulose substrates (Ding et al., 2012; Luterbacher et al., 2015; Donaldson and Vaidya, 2017). Ding et al. (2012) studied the localization of labeled enzymes during the degradation of different cell types in corn stover stems. These authors used Raman scattering to show lignified vs. non-lignified cell walls and light microscopy to perform real-time imaging of the morphological changes. They reported that enzymes did not bind to the lignified cell walls and that different patterns of cell wall deconstruction were observed according to the tissues and to the source of the enzyme mixtures. Donaldson and Vaidya (2017) quantified the spatial distribution of bound enzymes relative to lignin and cellulose in steam-exploded pine fiber by measuring the co-localization of enzymes, lignin and cellulose. They found a moderate correlation between the enzyme distribution and the cell wall histochemistry and a random association with lignin suggesting non-productive binding. They concluded that accessibility was a major determinant of enzyme binding compared to the biochemical composition. In a native substrate, Luterbacher et al. (2015) tracked both fluorescent labeled enzymes and the structure of the autofluorescent biomass during hydrolysis. By comparing hardwood and switchgrass with and without pretreatment, they concluded that enzymes bound predominantly to areas that had lost their original structure and exhibited low lignin fluorescence. They quantified the enzyme amounts by measuring the fluorescence intensities and showed that bound enzymes increased rapidly and then remained fairly constant throughout the hydrolysis. In all these studies, the enzymes had to be labeled.

All these papers reveal the great complexity of the elucidation of the recalcitrance of lignocellulosic biomass to degradation. Tracking both the enzymes and the evolution of the biochemical composition of cell walls is a significant challenge. Multimodal imaging is necessary for this purpose. In the present work, we used fluorescence imaging and infrared microspectroscopy to develop time-lapse imaging of the enzymatic degradation of lignocellulosic biomass without any labeling of the enzymes or the cell walls. The SOLEIL synchrotron facility is inherently a multimodal platform that has developed low-energy imaging beamlines. The synchrotron radiation provides deep-UV illumination, enabling protein autofluorescence (Jamme et al., 2013). After excitation at 275 nm, enzymes are visible due to the tryptophan and tyrosine autofluorescence. Using this facility, Tawil et al. (2011) and Jamme et al. (2014) followed the 3D real-time enzymatic degradation of a starch granule by an α-amylase. Using the same excitation wavelength, the autofluorescence of phenolic compounds in lignocellulosic plants makes cell walls visible over 380 nm (Jamme et al., 2013), providing morphological information. Polysaccharides and lignin can be characterized by mid-infrared microspectroscopy (Chazal et al., 2014). Using the synchrotron source, a spatial resolution compatible with the sizes of the cell and cell wall (10–50 and 1–5 μm, respectively) can be attained with a good signal-to-noise ratio (Allouche et al., 2012). We took advantage of a microfluidic device developed by the synchrotron SOLEIL SMIS beamline (Frederick et al., 2012; Sandt et al., 2013) for recording time-lapse infrared spectra during enzymatic hydrolysis.

Maize stems were considered as a model and application plant representative of poaceae lignocellulosic biomass. The experiments focused on the degradation of different cell types in parenchyma and vascular bundles near the rind by a cellulase cocktail. In this region of the stem, contrasting behaviors of degradation and lignification of the cell wall have been reported (Jung and Casler, 2006; Zhang et al., 2013). The following questions were addressed: the localization of the enzymes, the determination of the kinetics of degradation for different cell types, and the quantification of the biochemical modifications in degraded and recalcitrant cell walls.

## Materials and methods

### Plant material

Maize genotype F2 plants were grown at Lusignan (INRA, Unité de Génétique et d'Amélioration des Plantes Fourragères, France). Ear-bearing internodes were collected at the female flowering stage of development. Small cubes were cut from 5-mm-thick cross-sections sampled from the middle of the internodes. Cubes were taken in order to observe parenchyma and vascular bundles under the rind. The cubes were fixed in FAA (formaldehyde 40%, acetic acid, ethanol) for 24 h at 4°C, dehydrated with a graded aqueous ethanol series and then embedded in paraffin (Tissue Processor Leica 1020, France). Cross-sections of 16 μm thickness were obtained using a microtome (Microm HME 340E, MM France). The sections were treated with a histochoice clearing agent (Sigma H2779) solution to remove paraffin, and then with an α-amylase (Sigma A3176, 1 mg/mL, phosphate buffer 0.02 M pH 6.8, 6 h at 37°C) and a protease (Sigma P5380, 10 mg/mL, phosphate buffer 0.1 M pH 7.2, 3 h at 37°C).

### Maize stem cell types

**Figure 1:**
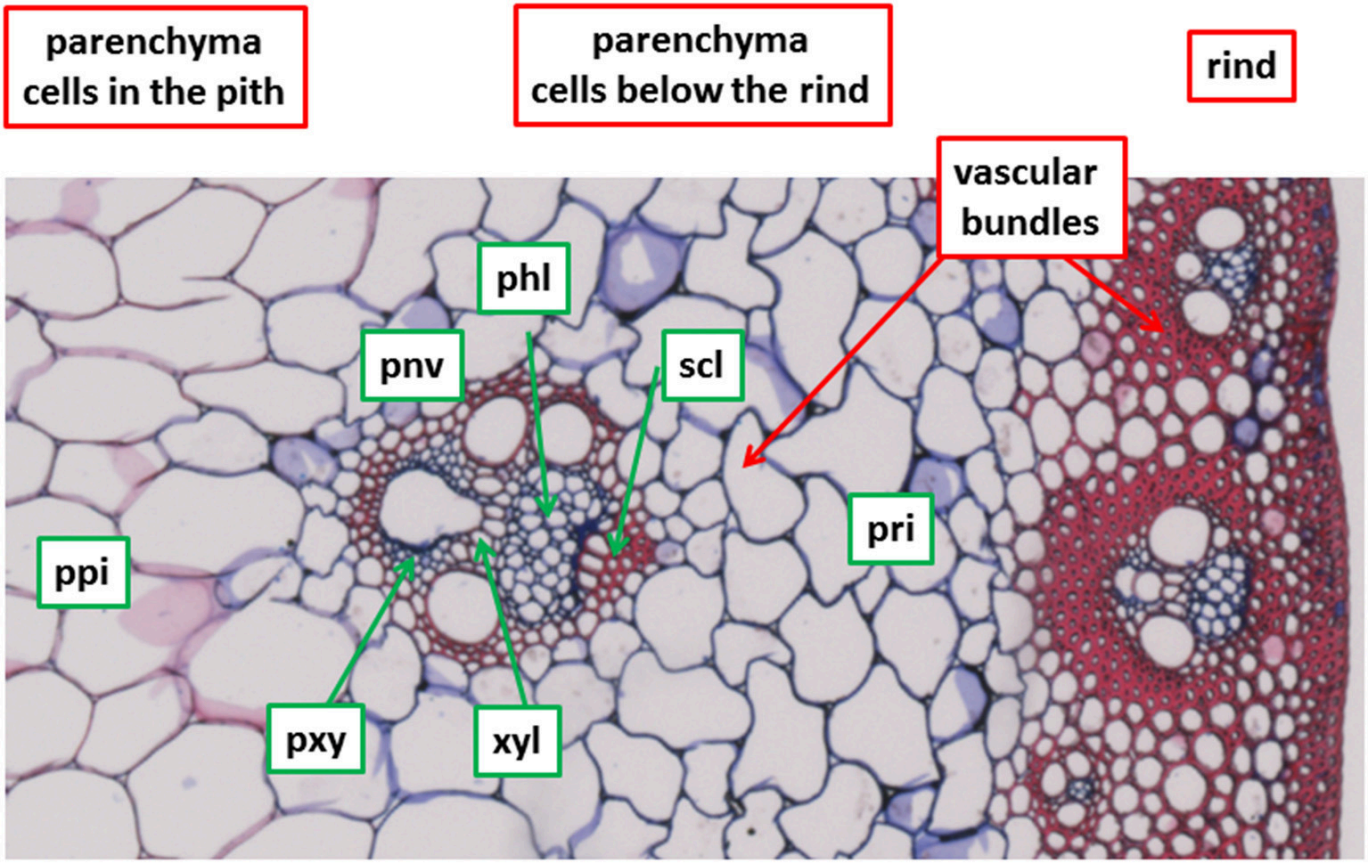
Selected cell types. Fasga staining as described in Méchin et al. (2005). Lignified cell walls appear in red, non-lignified cell walls appear in blue. Field of view: 1,190 × 595 μm2. See Table 1 for cell type codes.

Seven cell types that differ in degradability properties (Jung and Casler, 2006) were *a priori* considered to describe and quantify cell wall degradation (Table 1). They are shown in Figure 1 for a stem section after Fasga staining as described by Méchin et al. (2005). Three regions were distinguished: the rind made of epidermis, hypodermis and parenchyma cells with thick lignified cell walls and small embedded vascular bundles; the parenchyma region below the rind with non-lignified cells and including vascular bundles; and the parenchyma region corresponding to the pith, with large lignified cells and including vascular bundles. In the present paper, three parenchyma cell types were chosen: parenchyma in the pith (ppi), parenchyma below the rind (pri) and parenchyma near vascular bundles (pnv). Four other cell types were found in a vascular bundle selected in the parenchyma region below the rind: sclerenchyma sheath (scl), xylem fibers (xyl), phloem (phl), and xylem parenchyma (pxy). Among those cell types, the sclerenchyma sheath of the vascular bundle (scl), xylem fibers (xyl), and the parenchyma in the pith (ppi) have been shown to be lignified (Méchin et al., 2005).

**Table 1:** Cell types, codes, known recalcitrance toward cellulolytic enzymes (Jung and Casler, 2006).

**Codes**	**Cell types**	**Plant tissue**	**Recalcitrance to degradation**
pri	Parenchyma close to the rind	Parenchyma cells	Degraded
pnv	Parenchyma close to vascular bundle	Parenchyma cells	Degraded
ppi	Parenchyma between vascular bundles in the pith.	Parenchyma cells	Recalcitrant
scl:	Sclerenchyma sheath		Recalcitrant
xyl	Xylem fibers	Vascular bundle	Recalcitrant
phl	Phloem		Degraded
pxy	Xylem parenchyma		Degraded

### Cell wall-degrading enzymes

The cell wall enzymatic digestion was performed using the commercial cellulase Celluclast® 1.5 L (batch n° CCN03123) derived from the fungus Trichoderma reesei (Novozymes A/D, Bagsvaerd, Denmark). The enzyme preparation was desalted on a PD-10 column (GE Healthcare Bio-Sciences AB, Uppsala, Sweden) and the activities were determined on model substrates (Table 2). The preparation was diluted to have an enzyme activity on carboxymethylcellulose of 0.67 nkat/mL.

**Table 2:** Enzymatic activities in desalted celluclast preparation.

**Substrates**	**Activities (nkat/mL)**
Carboxymethylcellulose	1,473
Mix linkage β-glucan	1,558
Water-extractable arabinoxylan	752
Water-unextractable arabinoxylan	369
p-nitrophenyl glucoside	126
p-nitrophenyl xyloside	59

### Synchrotron fluorescence imaging

The SOLEIL-DISCO beamline and the imaging microscope setup has been fully described in Giuliani et al. (2009) and Jamme et al. (2010, 2013). The so-called Telemos device is a modified full field microscope (Axio Observer Z1, Carl Zeiss GmbH, Germany) coupled to the monochromatized synchrotron beam. The microscope is equipped with a back-illuminated CCD camera (Pixis BUV, Princeton Instrument, USA) that allows 65536 gray levels to code fluorescence intensity. Two objectives were used: 10× (NA 0.2) and 40× (NA 0.6) Ultrafluar Zeiss (Carl Zeiss GmbH, Germany) that provide fields of view of 1,116 × 1,116 and 292 × 292 μm2 with pixel sizes of 1.092 and 0.2853 μm, respectively. The excitation wavelength was set to 275 nm. A dichroic mirror at 300 nm (Omega Optical Inc., USA) and two bandpass filters were used for multispectral image acquisition: Emission at 327–353 nm (Semrock, Rochester, USA) to recover the tryptophan and tyrosine autofluorescence and therefore obtain images of the enzymes without any labeling, and Emission 420–480 nm (Semrock, Rochester, USA) to acquire cell wall images due to the autofluorescence of the phenolic compounds. The acquisition time was set to 15 s per image at 10× magnification for both filters and to 17 and 12 s for the 327–353 and 420–480 nm emission filters, respectively, at 40× magnification.

**Figure 2:**
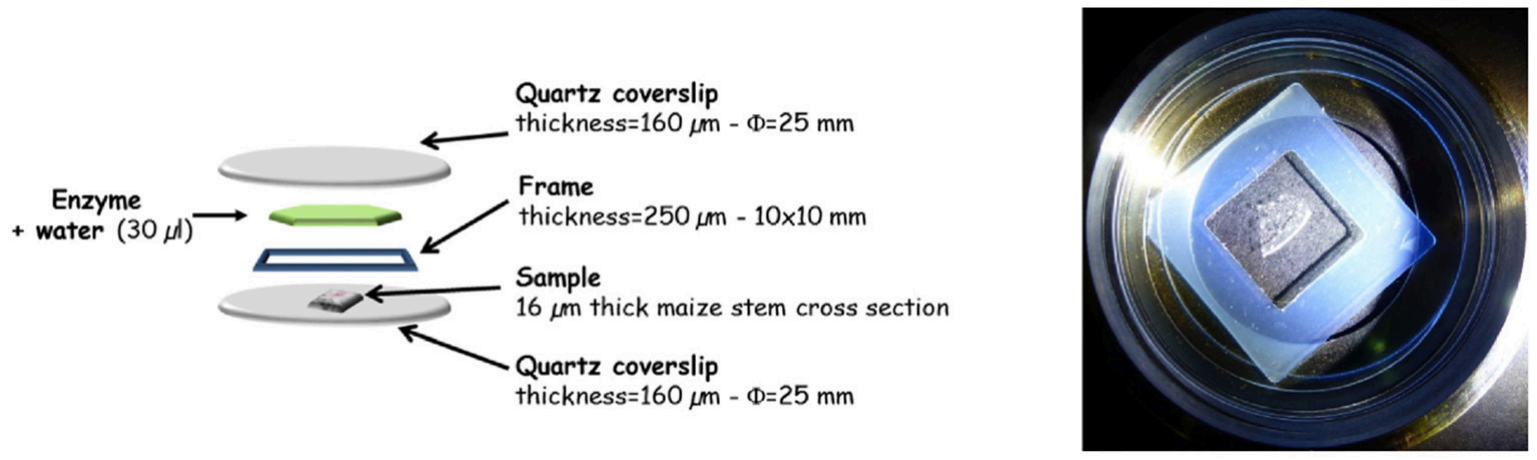
Design of the cell for fluorescence imaging of the enzymatic degradation at the DISCO beamlines.

The 16-μm-thick sections were placed on a circular quartz coverslip (Circular-Qtz 25.0 Ref. R525000, Esco optics, NJ 07438, USA). A cell was set up to perform the enzymatic degradation (Figure 2). A frame with a 250 μm thickness and a 10 × 10 mm2 size (Ref AB0576 Gene Frame®, Thermo Scientific, France) surrounded the section. Then, 30 μL of diluted enzyme was poured inside, and the cell was closed by a second circular quartz coverslip. The reactions were carried out at room temperature. Time-lapse images were acquired every 6 min over a total of 60-75 min for each experiment.

### Synchrotron FT-IR imaging using a microfluidic device

Mid-infrared spectra were acquired at the synchrotron SOLEIL-SMIS beamline using an FT-IR microspectrophotometer coupled to the synchrotron source (spectrometer Thermo Nicolet 5700 combined with a Nicolet Continuum XL microscope - Thermo Fisher Scientific, WI, USA). The microscope was operating in confocal mode using a double-path aperture size of 12 microns, a 32× infinity corrected Schwarzschild objective (NA = 0.65) and a matching 32× condenser. The microscope was equipped with a liquid nitrogen-cooled mercury cadmium telluride (MCT-A) detector (50 μm pixel size).

**Figure 3:**
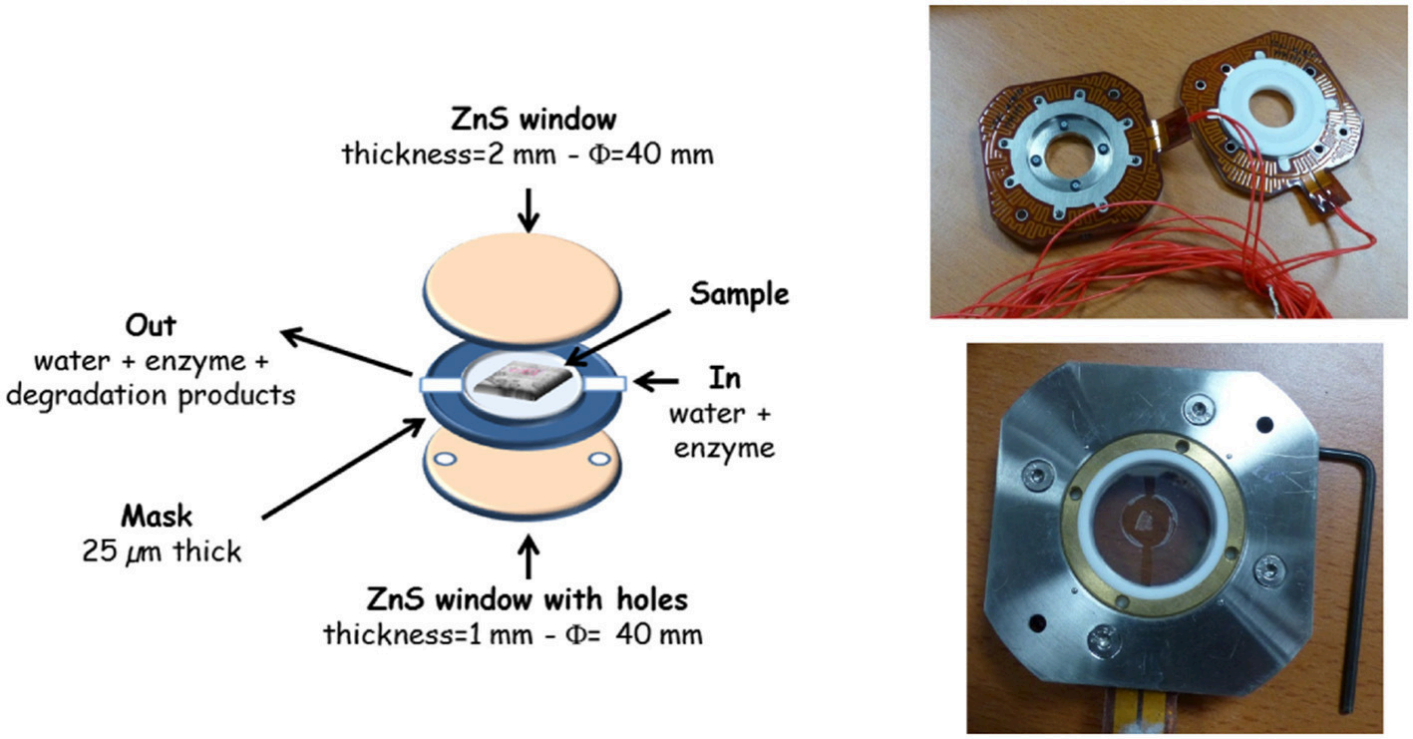
Microfluidic cell for synchrotron infrared imaging at the SOLEIL-SMIS beamline.

The enzymatic degradation was performed under the microscope using the microfluidic device developed at the SMIS-beamline (Frederick et al., 2012; Sandt et al., 2013). The device body is made of stainless steel (Figure 3). Two ZnS windows, 40 mm in diameter (Crystal GmbH, Berlin, Germany), were built specifically for the experiments. The bottom one was 1-mm thick with four drilled holes, and the top one was 2-mm thick and lacked holes. ZnS windows were chosen to allow the analysis of the spectral sugar fingerprint region. Spacer masks were cut in a 25-μm-thick film of ethylene tetrafluoroethylene copolymer (ETFE) (GoodFellow, Lille, Fr. Ref FP361025). The spacer masks acted as a sealant between the two ZnS windows and ensured fluid circulation and the positioning of the sample (Figure 3). The whole cell volume was ~4 μL. The mounting of the cell was performed as follows. A 16-μm-thick maize stem section was placed on the 2-mm-thick ZnS window in the place defined by the mask. The second ZnS window was then placed, and the two parts of the cells were screwed together. The microfluidic cell was connected to a high-precision low flow rate syringe infusion pump (WPI AL 1000, Hertfordshire, UK). Water was delivered until all the air bubbles were removed. The enzyme solution was then delivered with a constant flow of 2 μL/min. The enzymatic degradations were performed at room temperature.

All spectra were obtained using a masking aperture of 12 × 12 μm2 in order to recover a sufficient signal-to-noise ratio. The condenser was adjusted before each experiment. Spectra were collected in the 4,000–800 cm^−1^ infrared range at a resolution of 8 cm^−1^ with 128 co-added scans. The aqueous medium (water alone or water + enzymes) was acquired as a background.

Six experiments with enzymes were performed. For each experiment, brightfield images were acquired using a CCD camera (Sentech ST-C83USB-AS, Aegis Electronic Group, USA) and automatically stitched together using OMNIC 8.2 Atlus 8.1 software (Thermo Fisher Scientific, WI, USA) to form a mosaic image. From the mosaic image, 4 points were selected, and time-lapse spectra were acquired every 6 min over a maximum of 60 min. The experiment was stopped after the degraded cell walls had fully disappeared. The experiment was retained for analysis when no noticeable sample drift was observed in the brightfield image. Because of the limited time for data acquisition at the synchrotron, no time-lapse experiment was performed without enzyme. Maps were nevertheless acquired without enzyme by selecting points on the cell walls in order to obtain spectra of the initial cell walls in the microfluidic cell. The coordinates of points on the mosaic images were automatically recorded with the spectra.

### Data analysis

#### Fluorescence image analysis

All the fluorescence images were pre-processed to remove the background and compensate for the illumination inhomogeneity using the principles described in Tomazevic et al. (2002) for shading correction:

(1) $$\begin{array}{r} \text{IMC}=\left(\text{IM}-\text{BKG}\right)/\text{ILL} \end{array}$$

where IM is the raw image, BKG is the additive background, ILL is the illumination inhomogeneity, and IMC is the corrected image. A black camera image was taken as the background, and the illumination was estimated from the enzyme image using a low-pass Gaussian filtering of the Fourier transform of the image. The enzyme and the cell wall fluorescence images were combined for visualization purposes to obtain an RGB image. The enzyme image was put into the red and green channels, while the cell wall image was put in the blue channel. Movies were built from the images acquired over time to visualize the dynamic evolution of the section during the enzymatic degradation.

The fluorescence intensities were measured to quantify the relative number of enzymes found on specific regions of the maize stem section. Regions corresponding to portions of cell walls were manually selected for different cell types, as shown in the Supplementary Data S1. Depending on the cell types, between 2 and 5 regions were manually selected. Additional regions corresponding to cell cavities were also considered. The intensities were measured according to time to establish kinetic curves. The intensities were compared using variance analysis by considering the cell types, the time and the experiments as factors depending on the hypothesis tested. Image processing and variance analyses were performed using the image processing and statistics and machine learning toolboxes from MATLAB (2015a)^1^ (The MathWorks, France).

#### Spectral analysis

The FT-IR spectra were pre-treated using homemade function written in MATLAB (2015a)^1^. After smoothing of size-5 spectral points, the 1,550–950 cm^−1^ region corresponding to the lignin and sugar region was selected. The region between 1,550 and 1,800 cm^−1^ could not be analyzed because of noisy signals due to the effect of using aqueous media as the background. The baseline was corrected by using linear segments with points *a priori* chosen for the whole spectral collection at 950, 1,180, and 1,550 cm^−1^. For each spectrum, the linear segments were adjusted by moving the end points to the nearest minimum. The 1,180–950 cm^−1^ region was considered to focus the analysis on the evolution of the polysaccharides during the degradation.

Spectra without enzyme were normalized so that the area under the spectrum was 1. Spectra taken during the degradation were normalized as follows: the maximum of the spectrum acquired at the beginning of the reaction was taken as a normalization factor for all the time-lapse spectra acquired at this point. The spectra were analyzed and compared using principal component analysis (MATLAB, 2015a)^1^. After the analysis, colored points corresponding to spectra without enzyme were drawn on the mosaic image by using spectral coordinates.

## Results

### Maize stem cell types

**Figure 4:**
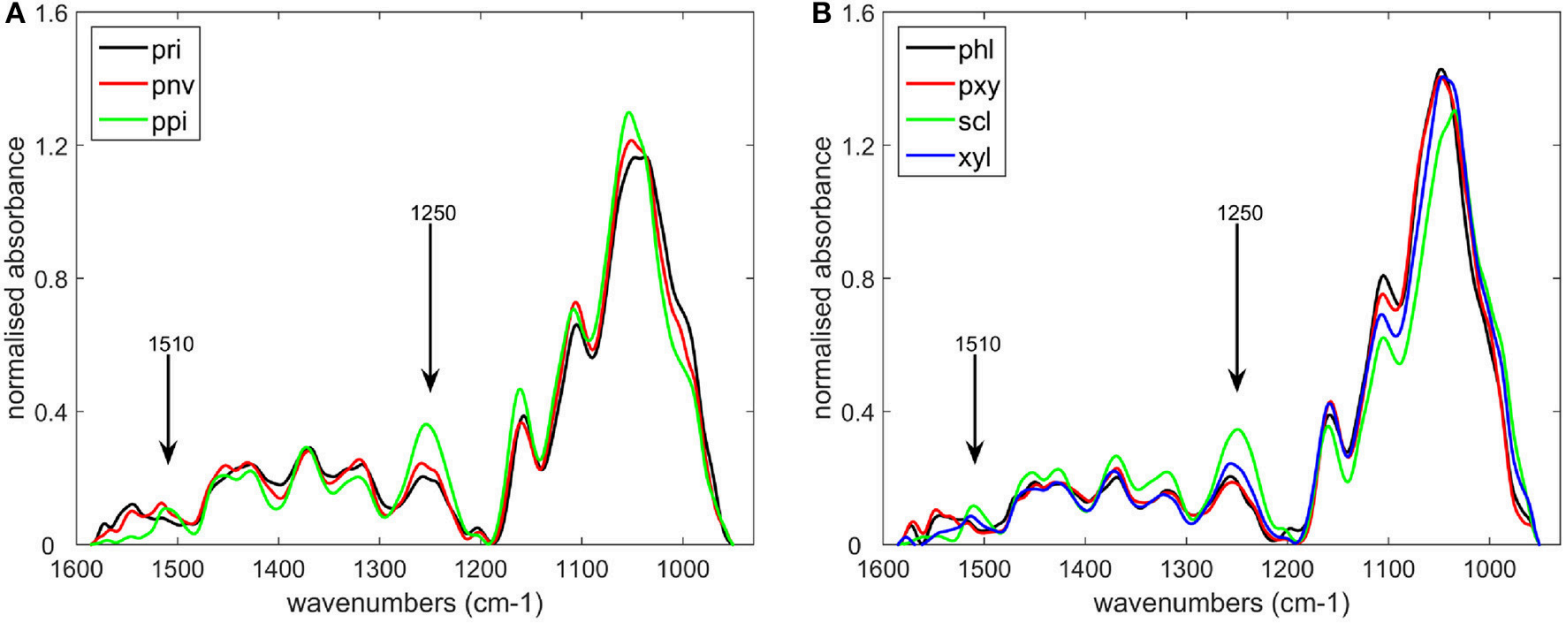
Average infrared spectra of the different cell types defined in Table 1. **(A)** parenchyma cell walls: near the rind (pri), near vascular bundles (pnv), in the pith (ppi), **(B)** cell types found in vascular bundles: phloem (phl), xylem parenchyma (pxy), sclerenchyma sheath (scl), xylem fibers (xyl).

The seven cell types described in Figure 1 and Table 1 were considered. Cell wall biochemical differences according to cell type were demonstrated by mid-infrared microspectroscopy using the microfluidic cell without enzyme. The average of five infrared spectra is shown for each cell type (Figure 4). By taking the aqueous medium as the background, relevant spectra could be acquired between 1,550 and 950 cm^−1^ in the aqueous medium without any significant deformation. In particular, no scattering artifacts or saturation effects were observed for the thickest cell walls, as confirmed by a single beam examination. In the case of lignified cell walls—parenchyma between vascular bundles in the pith (ppi) and sclerenchyma sheath of vascular bundles (scl)—the spectra showed a small absorption band at 1,510 cm^−1^, which could be assigned to an aromatic stretching vibration and is generally associated with lignin (Hergert, 1971; Faix, 1991; Chazal et al., 2014). They also showed a strong band at 1,250 cm^−1^ assigned to ester bounds (Hergert, 1971). These bands were lower in the spectra of parenchyma cell walls near the rind (pri), near vascular bundles (pnv), and of xylem parenchyma (pxy) and phloem (phl). The spectrum of xylem fibers (xyl) was intermediate. All the spectra exhibited absorption bands for polysaccharides between 1,200 and 950 cm^−1^ with a broad carbohydrate band at 1,035–1,051 cm^−1^ and partly resolved bands at 1,160, 1,110, 1,000–995 cm^−1^ (see also **Figure 9**) (Kačuráková et al., 1999; Maréchal and Chanzy, 2000; Kačuráková and Wilson, 2001; Robert et al., 2005). The significant overlap of these bands indicated a mixture of polysaccharides that varied between cell types. The phloem (phl) and xylem parenchyma (pxy) spectra exhibited a main band at 1,045 cm^−1^ with shoulders at 1,070, 1,000, and 980 cm^−1^, suggesting a higher contribution of hemicelluloses—mainly xylans—in these cell walls. In the spectrum of parenchyma between vascular bundles (pnv), two bands were observed at 1,035 and 1,053 cm^−1^ with a higher intensity at 1,035 cm^−1^. These wavenumbers correspond to those observed in cellulose spectra (Maréchal and Chanzy, 2000), suggesting that these cell walls may be enriched in cellulose.

**Figure 5:**
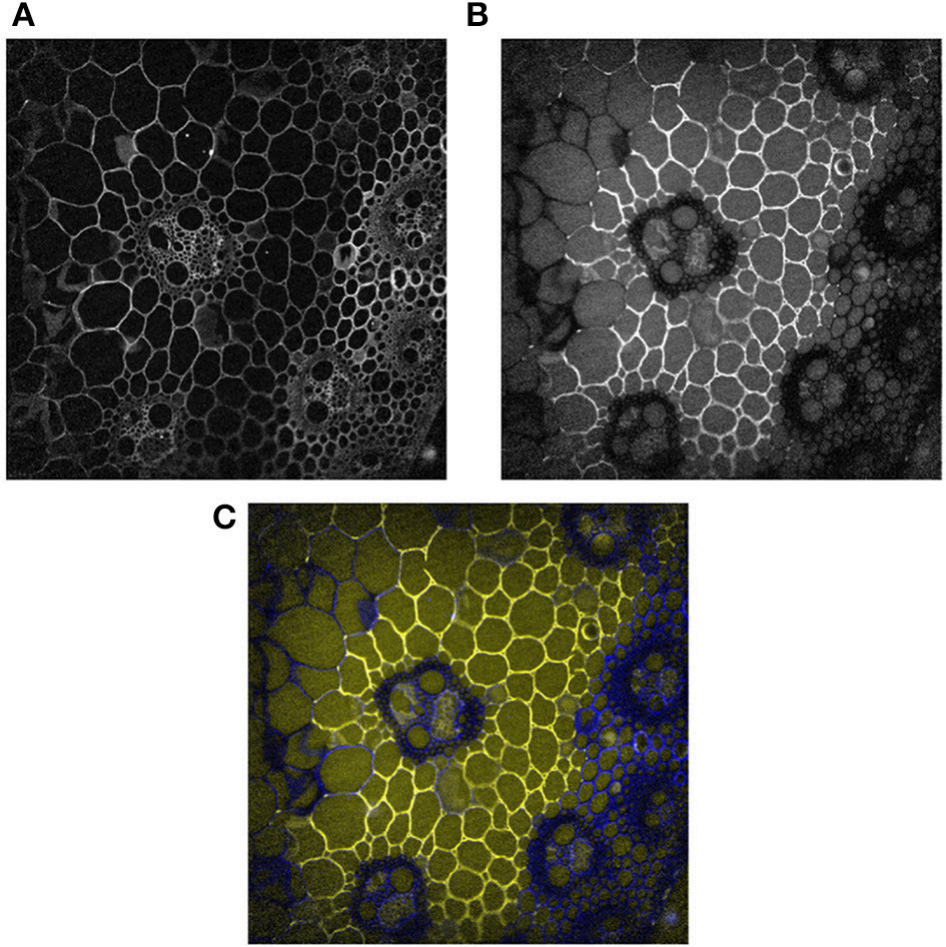
Example of fluorescence images 10 min after enzyme deposition. Magnification: 10×. The images were corrected for the background and illumination inhomogeneity. Field of view: 1,116 × 1,176 μm2. **(A)** Cell wall image: fluorescence between 420 and 480 nm. **(B)**: Enzyme image: fluorescence between 327 and 353 nm. **(C)**: Composite color images with the enzyme image in the red and green channels and the cell wall image in the blue channel. Blue, cell wall alone; Yellow, enzymes alone; White, co-localization of enzymes and cell walls.

Figure 5A shows one example of a fluorescence image acquired at 10× magnification between 420 and 480 nm after excitation at 275 nm. Under these conditions, lignins and hydroxycinnamic acids emit fluorescence (Jamme et al., 2013) and all the cell walls were fluorescent. Variations in the intensities were observed depending on the cell type. The highest intensities were found in the rind, for xylem fibers, xylem parenchyma and around phloem. The lowest intensities were observed for sclerenchyma sheath. We had already observed that the fluorescence intensity is lower in sclerenchyma cells than in phloem or xylem fibers (Allouche et al., 2012). In the present work, except for xylem fibers, lignified cell walls showed lower fluorescence intensities than other cell types.

### Localization of enzymes at the beginning of the reaction

Three experiments were performed at 10× magnification, two with enzymes and one without enzymes. Figure 5B shows the protein autofluorescence image acquired at 10× magnification between 327 and 353 nm after excitation at 275 nm and 10 min after enzyme deposition. As the proteins were removed from sections prior to enzymatic degradation, the fluorescence was ascribed to enzyme proteins, and the images were called enzyme images. A contrasted affinity of the enzymes according to the cell type was immediately observed. The enzymes were concentrated on parenchyma cell walls below the rind (pri) and seemed absent from sclerenchyma sheath (scl) and xylem fibers (xyl) in vascular bundles, parenchyma cells in the pith (ppi) and in parenchyma cells in the rind, i.e., from lignified cell walls.

The fluorescence intensities were measured in the enzyme images of the three experiments performed at 10× magnification. The average fluorescence intensities measured in the whole images were compared. Without enzyme, the average fluorescence intensities were very low (4.0 ± 2.6 counts), demonstrating that only enzymes contributed to the autofluorescence in the enzyme images. In what follows, the fluorescence intensities between 327 and 353 nm were therefore assumed to be proportional to the amount of enzyme. The intensities measured for the two experiments with enzymes differed, with average values of 84.7 ± 2.6 and 54.6 ± 2.0 counts, respectively. This can be ascribed to a different amount of enzymes deposited on the slides and to variations in the relative proportions of the cell types. The intensities were measured locally for specific regions corresponding to different cell types (Table 3). Cell cavities were also considered for parenchyma (pri) and (ppi) and were called (ipri) and (ippi). Variance analysis of these intensities showed a strong effect due to the cell type and the experiments. The difference in the average intensities of individual experiments did not affect the relative variations between the cell types, and the variations of the fluorescence intensities between cell types were subsequently interpreted as variations in the enzyme concentrations on the cell walls.

**Table 3:** Average fluorescence intensities measured in selected regions corresponding to cell types.

		**Average fluorescence intensity (counts)**	**Standard errors**
Vascular bundle	phl	61.3	3.0
	pxy	62.0	3.4
	scl	10.6	3.0
	xyl	22.8	3.4
Parenchyma	pri	96.0	3.0
	pnv	93.7	3.0
	ppi	13.7	3.0
Cell cavities	ipri	51.1	3.4
	ippi	49.6	3.4

*See Table 1 for the precise codes of cell types. The ipri and ippi regions were selected in cell cavities. All the other regions were selected on cell walls*.

The fluorescence intensities corresponding to the experiment shown in Figure 5 are given in Table 3. Three groups of intensities could be distinguished. In the cell cavities, only the enzyme solution was observed, and the intensity was ~50 counts. In the vascular bundle, the intensities for phloem and xylem parenchyma were slightly higher than in the cell cavities but were not significantly different. In contrast, parenchyma below the rind (pri) and parenchyma near the vascular bundle (pnv) showed higher fluorescence intensities (≈95 counts), while parenchyma in the pith (ppi), sclerenchyma sheath (scl) and xylem fibers (xyl) in vascular bundles showed very low fluorescence intensities (≈10–20 counts). Therefore, no enzyme fluorescence was observed on lignified cell walls, suggesting that no enzyme was present.

### Following the enzymes and the cell wall during the degradation

#### Fluorescence imaging

**Figure 6:**
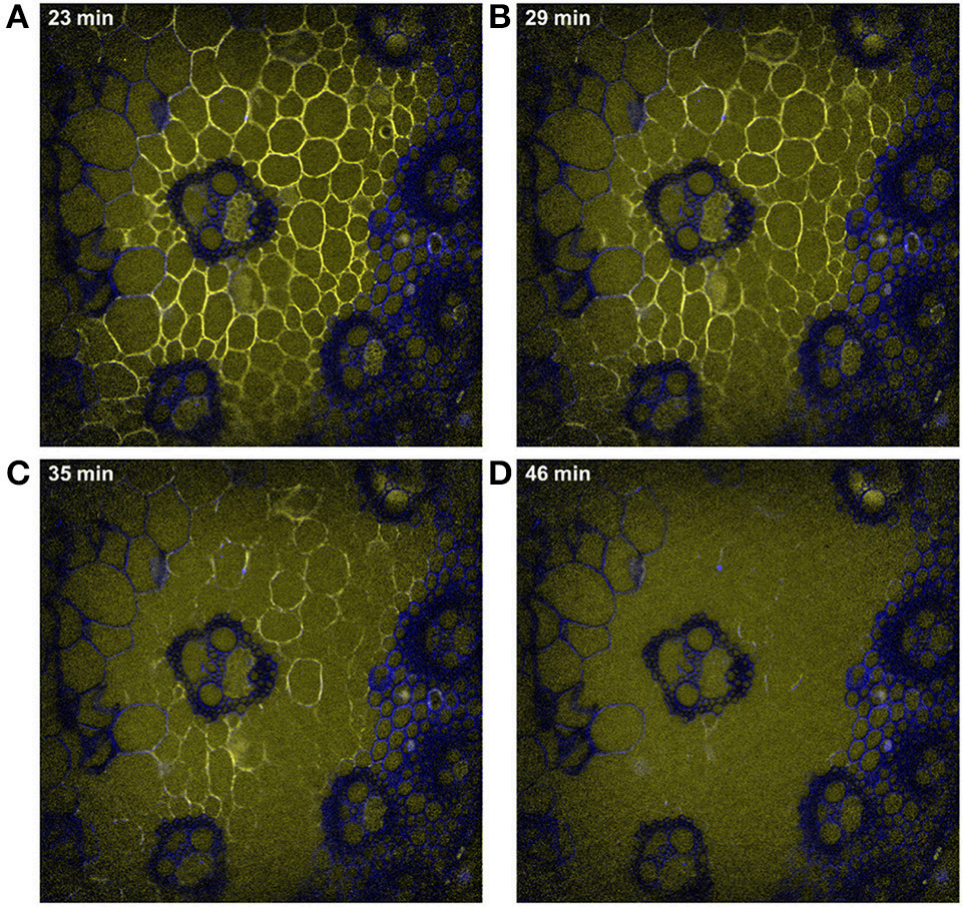
Examples of time-lapse fluorescence images corresponding to Figure 5. Magnification: 10×. The images were corrected for the background and illumination inhomogeneity. Field of view: 1,116 × 1,176 μm2. Blue, cell wall alone; yellow, enzymes alone; white, co-localization of enzymes and cell walls. **(A)** 23 min, **(B)** 29 min, **(C)** 35 min, **(D)** 46 min of hydrolysis.

Time-lapse fluorescence images were acquired to follow the cell wall degradation (Figure 6). Dynamic visualizations can be found in Supplementary Datas S2, S3 for the two experiments carried out with enzymes at 10× magnification. The images showed that only cell walls on which the enzymes were concentrated were degraded. No change could be seen in the lignified cell walls: enzyme fluorescence was not observed, and the cell walls remained unaltered.

In the example shown in Figure 6, the degraded cell walls had disappeared after 46 min. Looking at intermediate times, different rates of degradation were observed. Xylem parenchyma in the vascular bundle (pxy) disappeared after 23 min. In parenchyma cells, parenchyma near vascular bundles (pnv) seemed to disappear before parenchyma below the rind (pri), especially in the region between parenchyma in the pith (ppi) and the vascular bundle. In parenchyma below the rind (pri), the cells the closest to the rind were almost completely degraded after 35 min, while those half-way between the rind and parenchyma in the rind (ppi) were still visible. Similar comments could be made for the two experiments shown in Supplementary Datas S2, S3. In what follows, parenchyma below the rind was therefore separated into two sub regions: (pr1), the closest to the rind and (pr2), half way between the rind and parenchyma (ppi).

**Figure 7:**
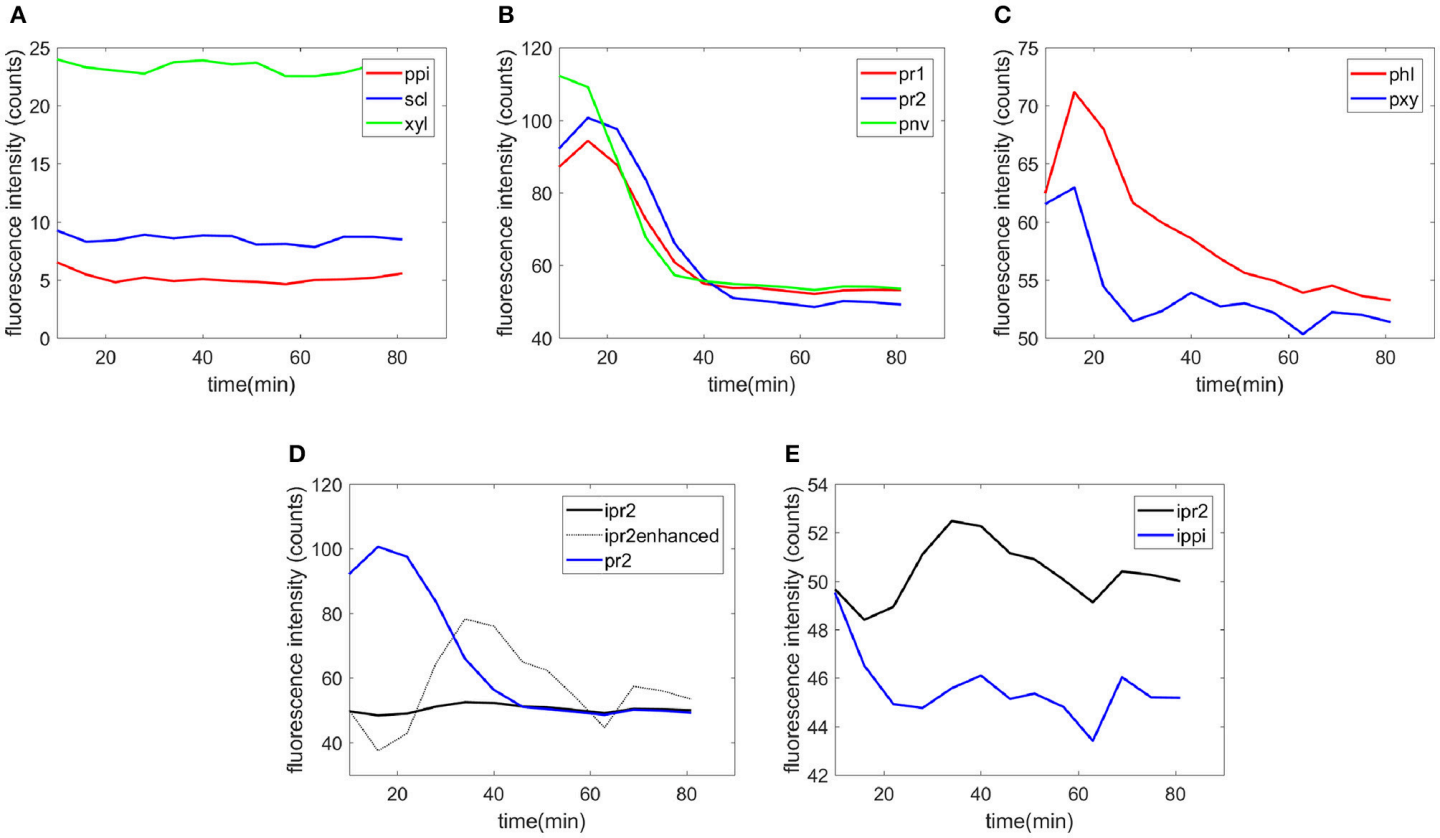
Examples of the time-course evolution of the fluorescence intensity. **(A)** Parenchyma in the pith (ppi), sclerenchyma (scl), and xylem fibers (xyl), **(B)** parenchyma below the rind (pr1 and pr2), parenchyma near vascular bundles (pnv), **(C)** phloem (phl), and xylem parenchyma (pxy), **(D)** fluorescence on the cell wall and in the cell cavity for parenchyma below the rind (pr2). The intensity of the cell cavity was enhanced for clarity by multiplying the intensity variations from the initial value by a factor of 10. **(E)** Comparison of the intensities in the cell cavity for parenchyma below the rind (ipr2) and in the pith (ippi).

Examples of the time-course evolution of the fluorescence intensity are shown for each cell type (Figure 7). No change in the intensity was observed for recalcitrant cell types [sclerenchyma (scl) and xylem fibers (xyl) in the vascular bundle and parenchyma in the pith (ppi), Figure 7A]. For the other cell types, a slight increase in enzyme fluorescence was observed at the beginning of the reaction (Figures 7B,**C**). This was interpreted as an intermediate increased concentration of enzymes on the cell walls. Then, the fluorescence intensity decreased to 50 counts, which corresponded to the fluorescence of the enzymes alone, providing evidence of cell wall degradation. Figure 7D compares the fluorescence intensities for the parenchyma cell wall (pr2) and cell cavity (ipr2). In the enhanced curve measured for the cell cavity, the increase of enzymes on the cell wall clearly occurred simultaneously with a decrease in the surrounding medium. In the cell cavity, the enzyme fluorescence decreased over 20 min, then increased with time until 40 min and finally decreased to reach a final value of 50 counts after 1 h. In contrast, in the cell cavities of recalcitrant parenchyma in the pith (ippi), a slight decrease in the fluorescence was observed over the first 20 min (Figure 7E).

The fluorescence quantification therefore provides measurements of the different rates of degradation according to the cell type. From the examples shown in Figure 7, it was shown that xylem parenchyma disappeared by 30 min. For phloem, a rapid degradation occurred during the first 30 min and then a slow decrease of the fluorescence was observed. To a lesser extent, Figure 7B also shows that parenchyma below the rind (pr2) disappeared after parenchyma close to the rind (pr1) and parenchyma near the vascular bundle (pnv).

**Figure 8:**
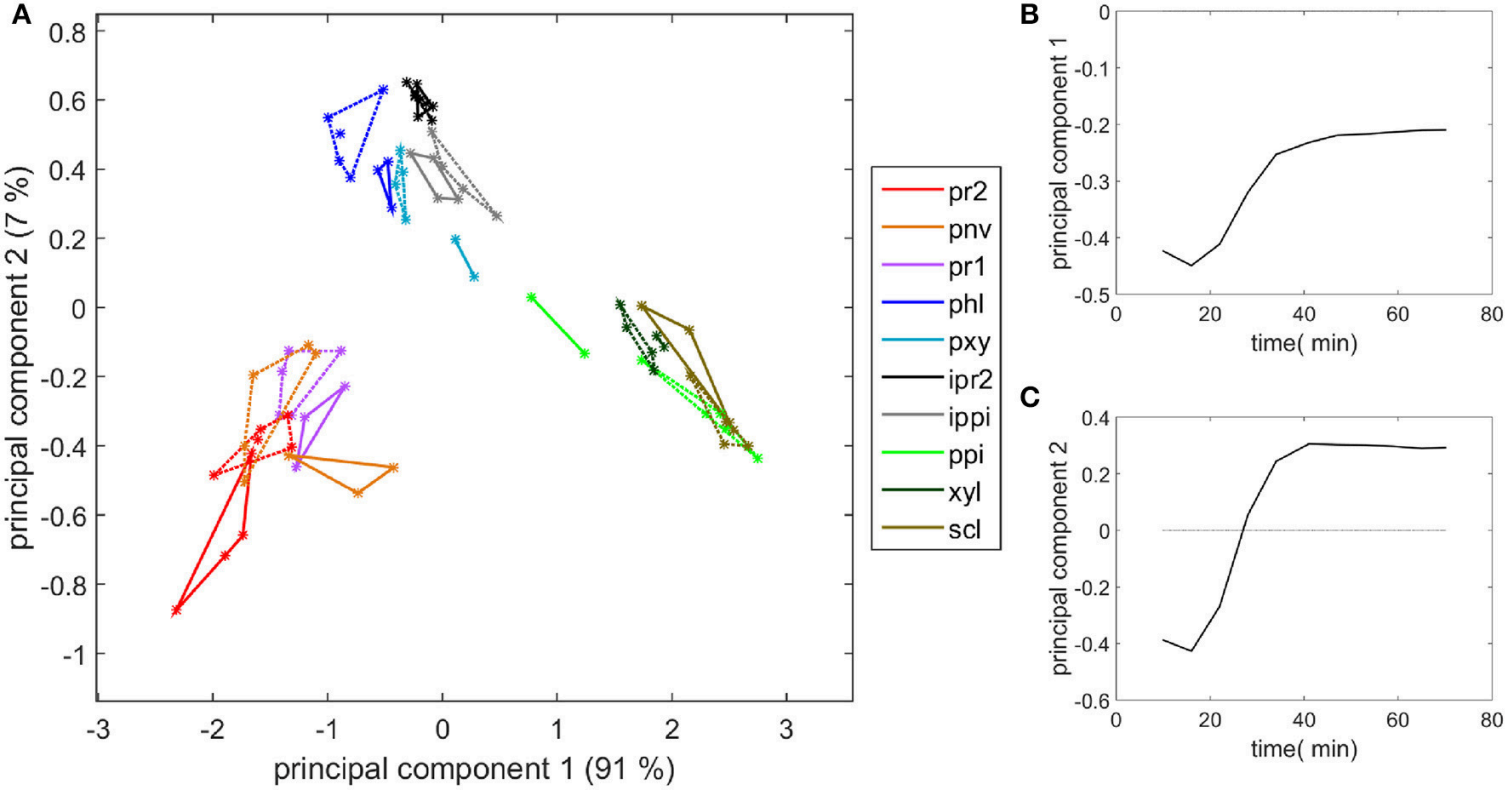
Principal component analysis of the fluorescence intensity evolution with time. **(A)** Scatter plot of components 1 and 2. See Table 1 for the code of cell types. (ipr2) and (ippi) refer to cell cavity measurements. Solid lines and dotted lines identify the two experiments with enzymes. **(B,C)** Loadings of components 1 and 2.

Similar measurements and comments can be made for the two experiments shown in the movies provided (Supplementary Datas S2, S3). To compare all the kinetic curves, the intensities were divided by the intensity observed for enzymes in the cell cavities, i.e., 50 and 75 counts for the two experiments, respectively. Principal component analysis was applied to the kinetics curves (Figure 8). The first principal component (91% of the total variance) described the differences in the intensities between the different cell types. Component 2 (7% of the total variance) described the contrast between the enzyme fluorescence at the beginning and at the end of the degradation. Consistent kinetic curves were observed for the two experiments (solid and dotted lines). Three main groups were observed. The first one corresponded to parenchyma cell walls (pr1), (pr2), and (pnv), for which enzymes were highly concentrated on the cell walls and that were totally degraded. The second group corresponded to the cell cavities (ipr2), (ippi), and to phloem (phl) and xylem parenchyma (pxy) in vascular bundles, for which enzymes appeared to be less concentrated on the cell walls. The last group corresponded to recalcitrant cell walls [sclerenchyma (scl), xylem fibers (xyl) and parenchyma in the pith (ppi)]. It should be noticed that, except for parenchyma near vascular bundle (pnv), the cell types did not overlap. This was ascribed to local heterogeneities in the cell wall degradation for this cell type (see Supplementary Datas S2, S3).

#### Microfluidic FT-IR imaging

**Figure 9:**
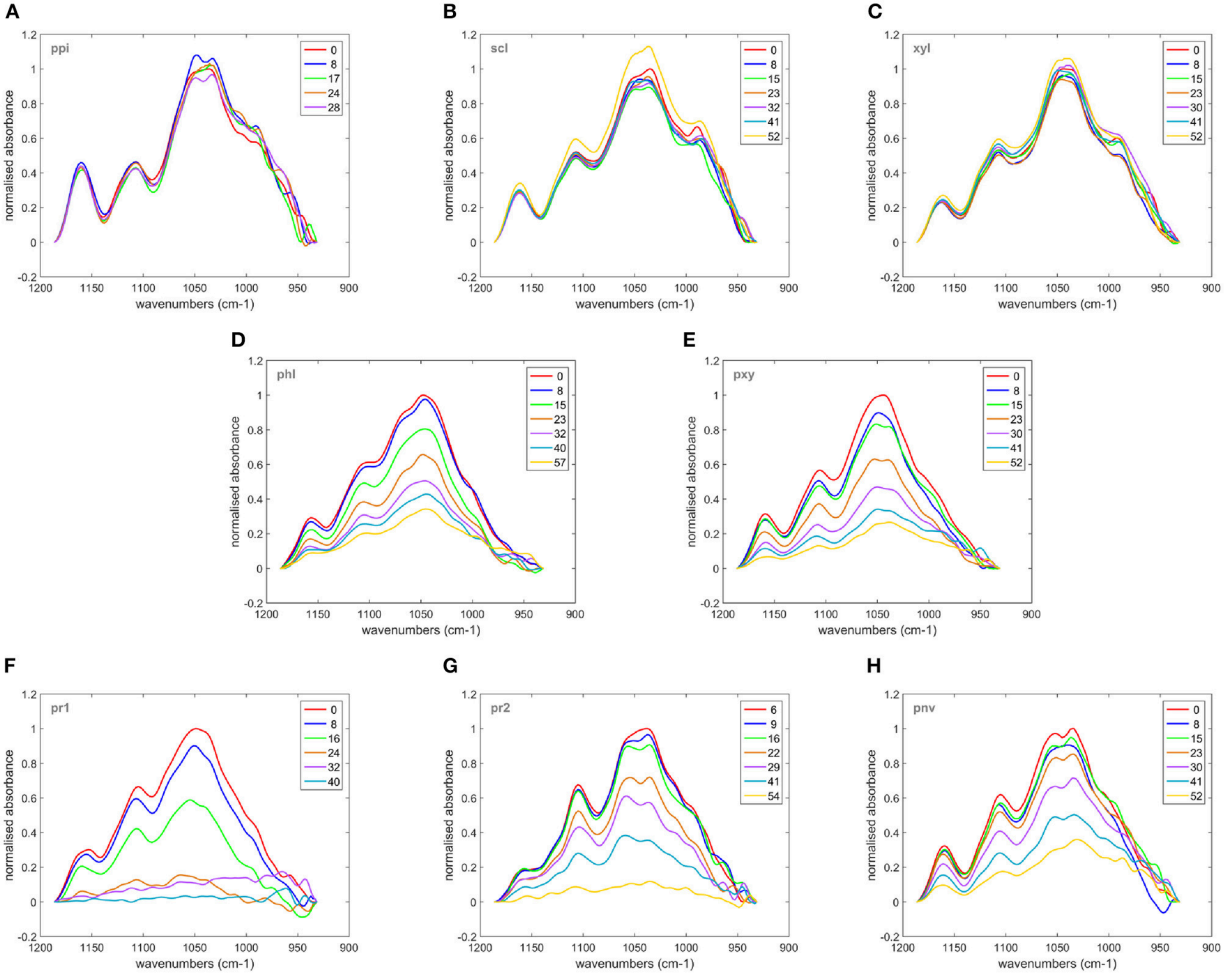
Time-lapse FT-IR spectra measured in the microfluidic cell. **(A)** Parenchyma in the pith (ppi), **(B)**: Sclerenchyma (scl), **(C)**: Xylem fibers (xyl). **(D)**: Phloem (phl), **(E)**: Xylem parenchyma (pxy) in vascular bundles, **(F,G)** Parenchyma below the rind: (pr1) and (pr2), **(H)** Parenchyma near vascular bundles (pnv). The colors indicate the reaction time in minutes.

**Figure 10:**
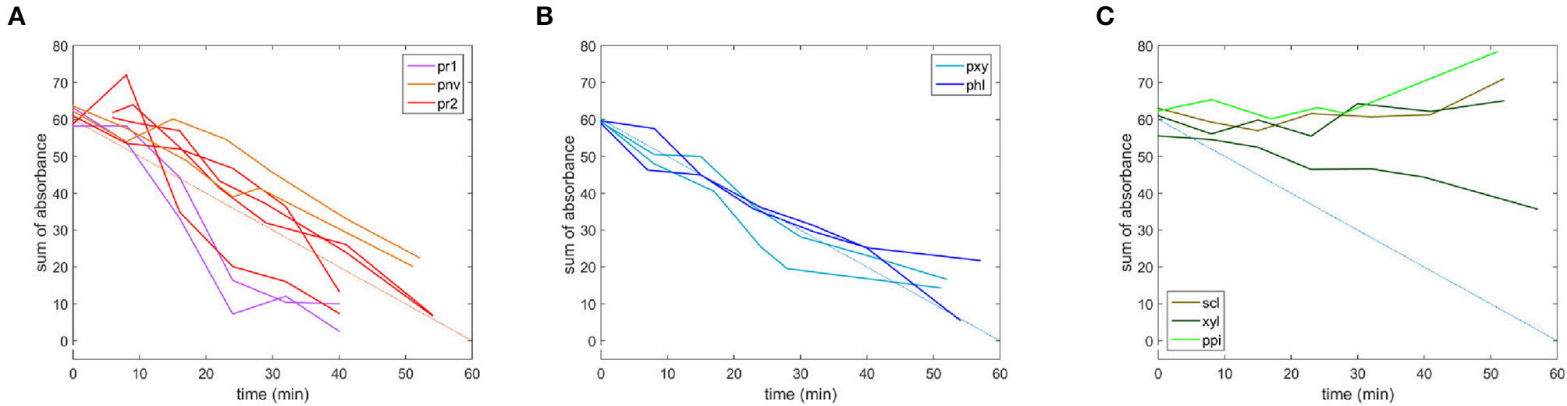
Microfluidic FT-IR time-lapse spectra with time, given as the sum of the absorbance. **(A)** Parenchyma below the rind (pr1) and (pr2), parenchyma near vascular bundles (pnv), **(B)** phloem (phl) and xylem parenchyma (pxy) in vascular bundles, **(C)** parenchyma in the pith (ppi), sclerenchyma (scl) and xylem fibers (xyl). An additional blue line was added to compare the degradation rates.

Fluorescence does not provide chemical information about the evolution of the cell wall polysaccharides during the enzymatic degradation. The infrared transparent microfluidic cell developed at the synchrotron SOLEIL SMIS beamline allowed the acquisition of time-lapse FT-IR spectra for 12 × 12 μm2 areas. The evolution of the sugar spectral fingerprint region between 1,180 and 950 cm^−1^ was considered. Figure 9 shows one example of a time-lapse spectrum for each cell type. In non-degraded cell walls, the spectra did not change during the reaction. In particular, no changes were observed for parenchyma between bundles in the pith (ppi). This result is consistent with the absence of enzyme fluorescence on the (ppi) cell walls and indicated that, given the sensitivity of infrared spectroscopy, the cell wall chemical composition was not modified. Similar comments can be made for sclerenchyma (scl) and xylem fibers (xyl). The apparent increase of intensity in the case of sclerenchyma after 52 min of reaction was due to slight displacements of the vascular bundle, likely modifying the amount of cell wall analyzed over the acquisition time. In degraded cell walls, the overall intensity decreased with reaction time, with different rates and extents of degradation. Full disappearance of the spectra was observed for parenchyma below the rind (pr1) and (pr2), and the degradation was more rapid for parenchyma (pr1). Figure 10 shows the evolution of the sum of the intensities of the time-lapse spectra with time for all the experiments. This confirms that parenchyma close to the rind (pr1) and (pr2) were almost totally degraded, in contrast to parenchyma near vascular bundle (pnv), phloem (phl) and xylem parenchyma (pxy). No evolution was observed for the other cell types. Different rates of degradation were observed depending on the cell type. Parenchyma close to the rind (pr1) was the most rapidly degraded. Parenchyma near vascular bundle (pnv), phloem (phl) and xylem parenchyma (pxy) were degraded slightly faster than parenchyma below the rind (pr2). For these cell types, the evolution of the infrared intensity was slower than expected from visual observation of the fluorescence images. For phloem, the results were consistent with the slow evolution of the enzyme fluorescence (Figure 7). For parenchyma (pnv), the delayed degradation of the polysaccharides was ascribed to heterogeneity in the cell wall composition.

**Figure 11:**
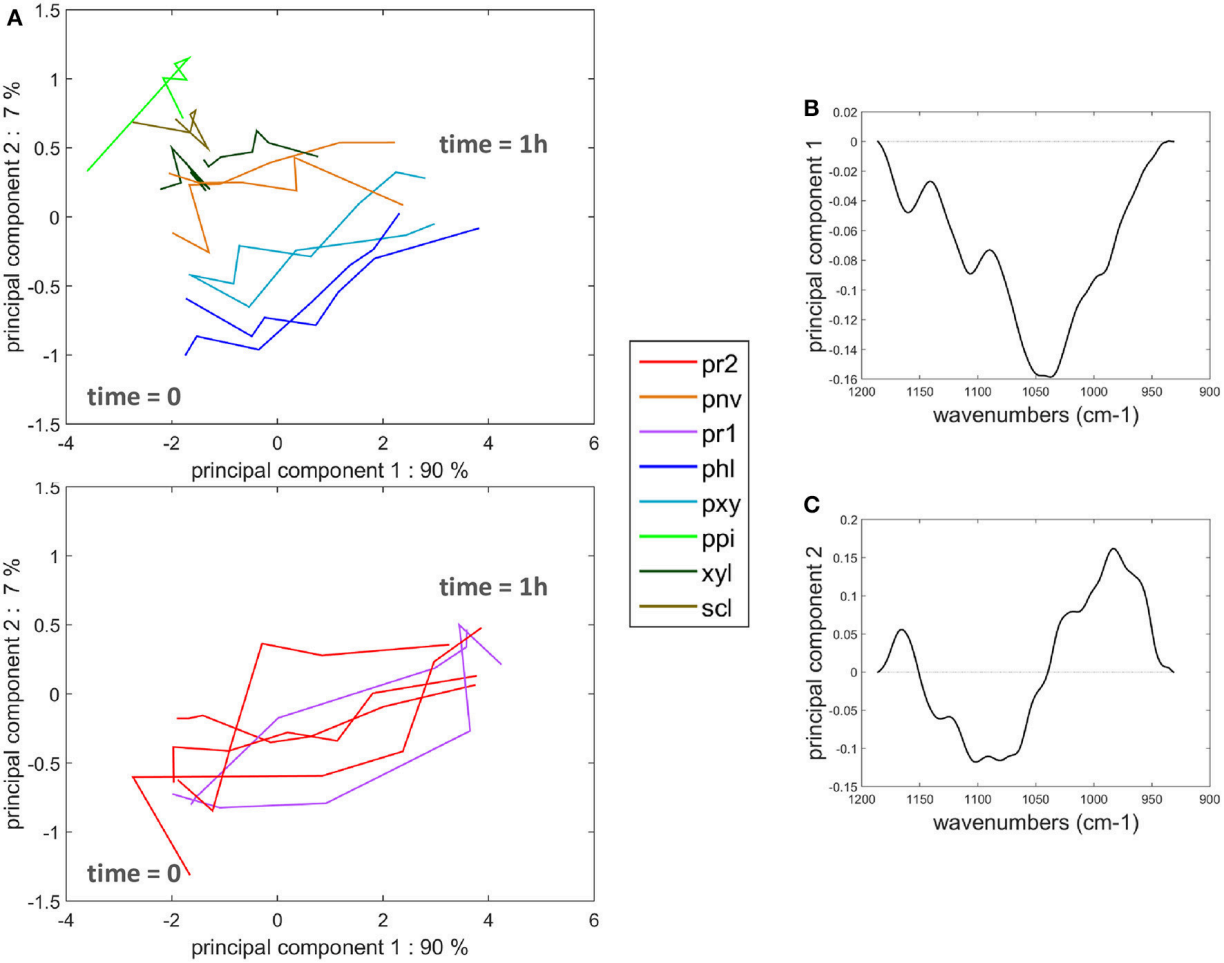
Principal component analysis of the microfluidic FT-IR spectra. **(A)** Scatter plot of components 1 and 2. See Table 1 for the code of cell types. Parenchyma below the rind (pr1) and (pr2) were drawn separately. **(B,C)** Loadings of components 1 and 2.

Principal component analysis was applied to the time-lapse spectra in the 1,180–950 cm^−1^ infrared region in order to compare the degradation behavior and biochemical composition, taking into account all cell types. The scatter plot defined by principal components 1 and 2 (90 and 7% of the total variance, respectively) and the corresponding loadings are shown in Figure 11. The first loading corresponded to an intensity absorbance loading. Negative bands were found at 990, 1,034, 1,042, 1,105, and 1,165 cm^−1^ that roughly corresponded to an average cell wall spectrum. Spectra at the beginning of the enzymatic degradation can be found on the left of the map. The second principal component separated tissues recalcitrant to enzymatic degradation from the other tissues. The loading plot revealed an opposition between positive bands at 985 and 1,165 cm^−1^ and negative bands at 1080, 1,100 cm^−1^. The two positive bands were on average higher in the spectra of the three recalcitrant cell types (Figure 9). The 1,165 cm^−1^ band has been assigned to p-coumarate in grass lignins (Chazal et al., 2014), and the 985 cm^−1^ band has been found in low-substituted xylans (Robert et al., 2005). The negative bands are commonly found in polysaccharides (Kačuráková and Wilson, 2001).

**Figure 12:**
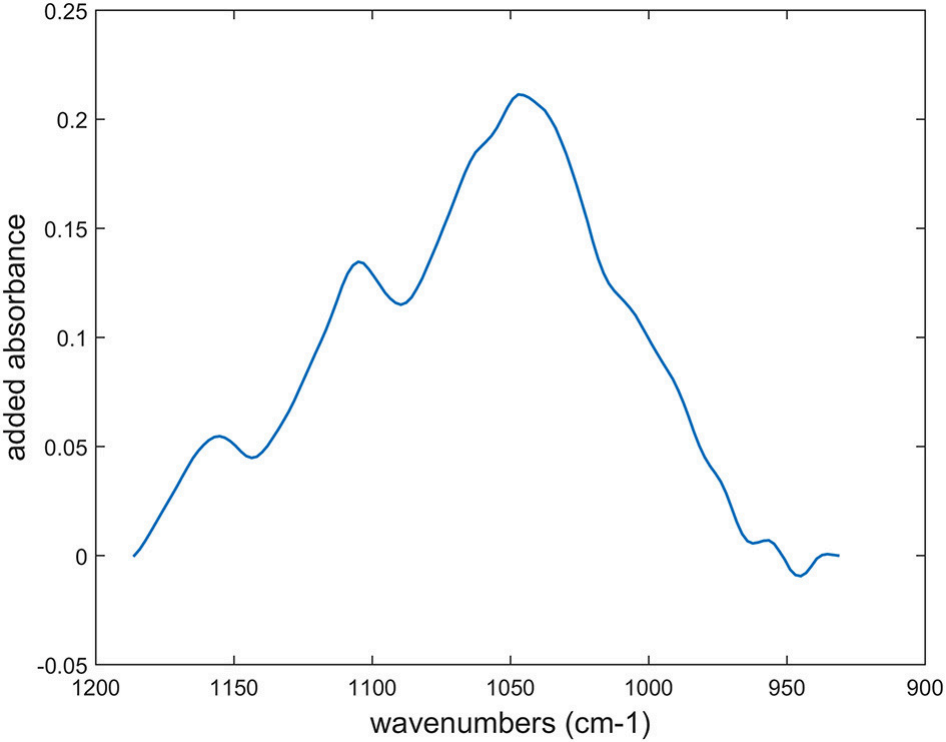
Sum of the difference spectra after area-normalization between time-lapse FT-IR spectra measured at the beginning of the reaction and after: 15 min for parenchyma below the rind (pr1) and 30 min for parenchyma below the rind (pr2), parenchyma near vascular bundles (pnv), phloem (phl) and xylem parenchyma (pxy) in vascular bundles. Individual difference spectra are shown in Supplementary Data S4.

The Celluclast preparation exhibited mainly cellulolytic activity but also contained significant xylanase activity (Table 2). The first step of the reaction was investigated. Spectra acquired after 30 min of enzymatic degradation were subtracted from the initial spectra for parenchyma below the rind (pr2), parenchyma near vascular bundles (pnv), phloem (phl), and xylem parenchyma (pxy). To account for the different rates of degradation, spectra acquired after 15 min were taken for parenchyma below the rind (pr1). For all tissues, a band was observed at 1,045 cm^−1^ in the difference spectra (Supplementary Data S4). Depending on the cell walls, cellulose bands at 1,030 and 1,060 cm^−1^ could also be observed. The difference spectra were area-normalized, and their sum was assessed for the 12 degraded cell walls (Figure 12). The resulting spectrum showed a main polysaccharide band at 1,045 cm^−1^, generally assigned to xylans, and although the enzymatic preparation was designed to be rich in cellulase activities, our results showed that xylans, i.e., hemicelluloses, were on average degraded prior to cellulose.

### Cell wall heterogeneity

The time-lapse fluorescence and microfluidic FT-IR imaging showed consistent results about the cell wall biochemical composition, enzyme localization, and cell wall degradation. However, some local heterogeneity was observed. In particular, repetitions of the experiments showed large variability for parenchyma below the rind (pr2) (Figures 10, 11). Discrepancies were also found between fluorescence and FT-IR analyses for parenchyma near vascular bundle (pnv).

**Figure 13:**
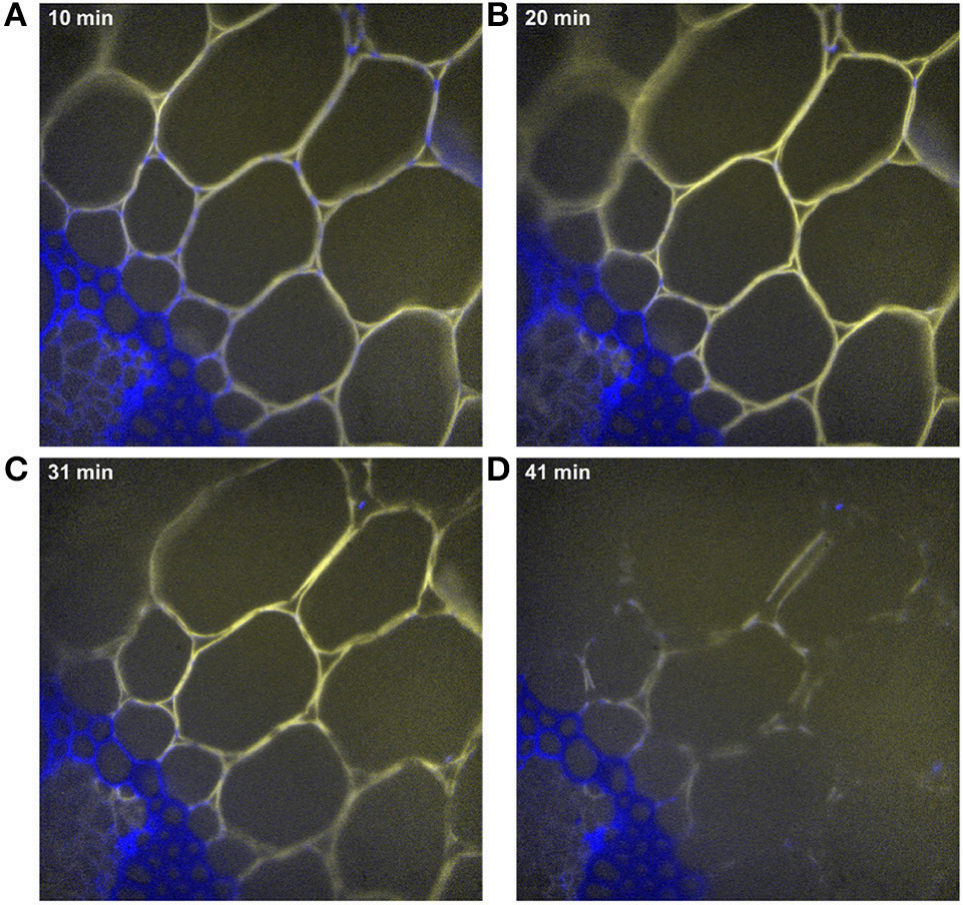
Examples of time-lapse fluorescence image for parenchyma below the rind (pr2). Magnification: 40×. The images were corrected for the background and illumination inhomogeneity. Field of view: 292 × 292 μm2. Blue, cell wall alone; yellow, enzymes alone; white, co-localization of enzymes and cell walls. **(A)** 10 min, **(B)** 20 min, **(C)** 31 min, **(D)** 41 min of hydrolysis.

Additional experiments were performed to investigate these results. The enzymatic degradation was observed by autofluorescence imaging at higher magnification (40×) (Figure 13 and Supplementary Data S5). This observation confirmed that, after 10 min of reaction, enzymes were absent from sclerenchyma cell walls and concentrated on parenchyma cell walls. On parenchyma cell walls, blue dotted points were observed mainly on cell junctions, showing that the enzymes were not evenly located. After 20 min, blue dotted points were less visible, showing that the enzymes progressively bound to those parts of the cell wall. After 30 min, adjacent cell walls started to dissociate and almost totally disappeared after 40 min. It should also be noticed that the rate of degradation was not faster for parenchyma near vascular bundles, i.e., connected to vascular bundles, than for the large parenchyma cells (pr2). In addition, the degradation was not homogeneous along the whole cell wall. The cell walls seemed to be fragmented in segments (Figure 13, 41 min).

**Figure 14:**
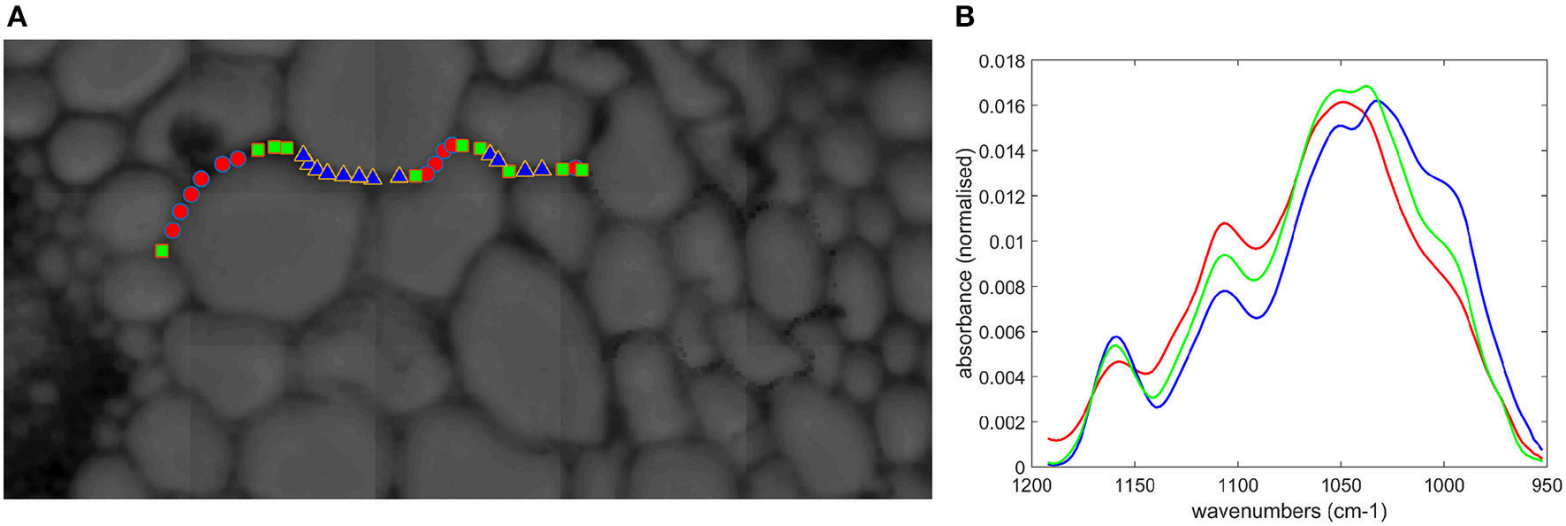
Microfluidic FT-IR spectra of parenchyma below the rind (pr2) without enzymes. **(A)** Selected points on the visible image. **(B)** Average spectra corresponding to the color symbols shown in the image.

FT-IR spectra were acquired along the cell walls of a few parenchyma cells (pr2) (Figure 14). The sugar region was analyzed by principal component analysis. A strong heterogeneity between spectra was observed, but it was possible to separate them into three groups. Their averaged spectra together with the localization of the points were computed (Figure 14). The bands at 1,160, 1,105, 1,053, 1,047, 1,035, 995 cm^−1^ were present with different relative intensities. In the blue group, the 995 cm^−1^ shoulder was high, together with the 1,160 cm^−1^ band. Together with the 1,035 and 1,053 cm^−1^ bands, this spectrum could be representative of cellulose (Maréchal and Chanzy, 2000). In contrast, the 990 and 1,160 cm^−1^ bands were low for the red group, and the maximum of the sugar band was observed at 1,047 cm^−1^. This spectrum may be representative of xylans (Kačuráková et al., 1999). The green group also exhibited a cellulose-like spectrum with similar bands to the blue group but with different relative intensities. Looking at the spatial distribution of the spectra, red, and blue points were adjacent, while the green points were more localized at cell junctions.

## Discussion

### Time lapse imaging of lignocellulosic biomass hydrolysis without any labeling

The objective of the work was to develop time-lapse imaging of the enzymatic degradation of lignocellulosic biomass by considering both enzyme localization and the biochemical evolution of the cell walls. Real-time imaging offers great potential for studying the impact of local biochemical variations on the binding of enzymes and the resulting time-lapse evolution (Ding et al., 2012; Luterbacher et al., 2015; Donaldson and Vaidya, 2017). Considering both the enzymes and the cell walls should highlight bottlenecks in the process, explaining the recalcitrance of cell walls to degradation. In the present work, the challenge was to image the enzymatic degradation of lignocellulosic biomass without labeling the enzyme or the cell walls. The idea was to take advantage of the contrasting spectral properties of enzymes and cell walls. Enzymes can be detected using protein autofluorescence in the deep UV even at low concentrations (Jamme et al., 2014), while plant cell wall polymers can be characterized by mid-infrared spectroscopy (Kačuráková and Wilson, 2001; Chazal et al., 2014). The synchrotron SOLEIL facilities allowed the design of the whole experiment. Deep UV illumination was possible, and the availability of a microfluidic infrared transparent cell made time-lapse infrared microspectroscopy possible. In addition, the synchrotron provided the spatial and temporal resolution necessary to study the mid-infrared spectral variability of cell walls at the cellular scale.

### Time lapse fluorescence image analysis

After excitation at 275 nm, both proteins and phenolic compounds autofluoresce (Jamme et al., 2013). Multichannel autofluorescence imaging using two channels allowed differentiation of the protein and the cell wall fluorescence so that they could be tracked during the reaction. The *cell wall image* showed the presence of lignified cell walls vs. cell walls rich in hydroxycinnamic acids. It also provided evidence for morphological changes. The *enzyme image* highlighted the presence or absence of enzymes on the cell walls. In addition to the qualitative observation of enzyme affinity toward non-lignified cell walls, the fluorescence intensity could be quantified by image analysis. The images had to be pre-treated to remove the background and illumination inhomogeneity inherent with synchrotron light in full field imaging.

Few studies have used quantitative fluorescence imaging to establish degradation kinetics. Luterbacher et al. (2015) have shown an increase in enzyme fluorescence at the beginning of the reaction by using labeled enzymes and confocal microscopy. In their work, the enzymes were fluorescent only when bound to the substrate. In our work, enzyme autofluorescence allowed the location of enzymes both on the substrate and in the surrounding medium. Consistent variations in the enzyme concentration were measured locally for cell cavities and their surrounding cell walls. The intensity decreased in the cavities while it increased on the cell wall. After degradation, the enzymes were released into the medium and the initial level of fluorescence was recovered. From a methodological point of view, this quantification demonstrates the power of enzyme autofluorescence imaging to reveal local variations in the enzyme concentration on the cell wall and inside cell cavities.

### Microfluidic mid-infrared time-lapse microspectroscopy of lignocellulosic cell wall hydrolysis

To our knowledge, only one publication has reported the use of a microfluidic device for tracking the biochemical evolution of plant material during enzymatic degradation by FT-IR microspectroscopy (Gierlinger et al., 2008). In the present work, 12 × 12 μm2 spatial resolution was attained together with a good signal-to-noise ratio due to the brightness of the synchrotron light source. It was possible to acquire spectra every 6 min. This spatial resolution and time step were relevant for revealing local variations and different degradation rates for the cell wall polysaccharides according to the cell type. The method, combined with multivariate data analysis, allowed definition and comparison of the kinetics of degradation, accounting for different patterns of degradation in non-lignified tissues. The analysis of the difference spectra at given times is also a promising way to relate the initial cell wall composition to specific enzyme action.

### Multiscale imaging

Significant heterogeneity is found in maize stem sections with different cell types and a heterogeneous spatial distribution of lignin at the macro scale (Zhang et al., 2013). Deep UV fluorescence imaging was performed at two magnifications. The 10× magnification had a 1 mm2 field of view that allowed observation of the different cell types in the region below the rind. This allowed comparison of lignified and non-lignified cell types. An unexpected variability was found in the parenchyma below the rind. The cells closest to the rind seemed to be degraded more rapidly in the fluorescence images, which was confirmed by the evolution of the mid-infrared spectra. This phenomenon has not been reported before and should be investigated further. In particular, the cell size seemed to be smaller for this region compared to the rest of the parenchyma below the rind. The cell wall composition and thickness should also be considered as part of interpreting the results.

Using 40× magnification, only a few cells of parenchyma below the rind (pr2) could be observed. Again, an unexpected variability was observed in the enzyme behavior. At the beginning of the reaction, the affinity of the enzyme depended on the micro-domains of the cell walls. Lower affinity was observed mainly in cell junction regions but also at some other points, resulting in a dashed effect. Enzymes progressively located in these regions. Variation in the cell wall composition according to the domain of the cell walls has been reported that could explain this behavior (Siqueira et al., 2011). In the present work, FT-IR imaging also showed strong variability in the initial biochemical composition, not only related to cell junction domains. This heterogeneity, reported here for the first time, will be further investigated.

### Enzyme localization

Enzymes were not observed on lignified cell walls. This result is consistent with the observations of Ding et al. (2012), who also reported the absence of enzymes on lignified maize stem cell walls. Luterbacher et al. (2015) also observed no binding of enzymes on particles rich in lignin, which kept their original structure after pretreatment. However, these authors used bovine serum albumin in the reactive medium to avoid non-specific enzyme binding. Working on pretreated biomass, Donaldson and Vaidya (2017) also found a low correlation between lignin and the signals of enzymes. They did not exclude non-specific binding but described it as a minor phenomenon. Non-specific adsorption of enzymes onto lignin has been mainly described with isolated lignins or lignin-rich residues (Haven and Jørgensen, 2013; Yarbrough et al., 2015; Zheng et al., 2016). This binding may be driven by hydrophobic interactions and electrostatic forces (Rahikainen et al., 2013; Sammond et al., 2014; Yarbrough et al., 2015). The properties of isolated lignin are indeed different from that of *in situ* lignin found in cell walls. The results observed with isolated lignin may not be able to be extrapolated to lignified cell walls. In accordance with the interpretation proposed by Ding et al. (2012) in the case of lignified secondary cell walls, our results suggest that lignin most likely acts as a physical barrier that restricts enzyme accessibility to the polysaccharide network.

In conclusion, at the beginning of the experiments, the enzymes seem to be excluded from lignified cell walls and to be concentrated in the parenchyma cell walls below the rind and around the vascular bundle, i.e., on cell walls that are generally degraded by cellulolytic enzymes. Intermediate concentrations were measured for phloem (phl) and xylem parenchyma (pxy) in the vascular bundle. A deeper analysis should also consider the cell wall thickness.

### Time-lapse evolution of the cell wall depended on the cell type

Fluorescence and mid-infrared quantification provided measurements of different rates of degradation according to the cell type. The fluorescence intensity quantifications showed that the enzymes were not evenly distributed, and their amount increased progressively on degradable cell walls. Different rates of degradation were found, and in particular, degradation was more rapid for parenchyma that are the closest to the rind. For parenchyma near vascular bundles, the heterogeneity observed did not lead to a precise conclusion. For phloem and xylem, the fluorescence evidenced a rapid evolution of enzyme concentration, while the mid-infrared spectroscopy indicated that residual material was present. However, the spatial resolution of the two techniques differed, with 12 × 12 μm2 in the case of the mid-infrared and ~1 μm2 in the case of the fluorescence.

Microfluidic FT-IR microspectroscopy allowed time-lapse tracking of local changes in the polysaccharides in cell walls during the degradation. For all the degraded cell types, hemicellulose degradation was found to occur prior to cellulose degradation using a Celluclast® preparation. This is consistent with the general scheme of the organization of the polymers in plant cell walls (McCann and Carpita, 2008), where cellulose microfibrils are embedded in a non-cellulosic polymer matrix, mainly composed of xylans in the case of poaceae.

Autofluorescence imaging and microfluidic infrared microspectroscopy were therefore shown to be appealing methods for a multimodal *in situ* labeling-free exploration of local and temporal variations during cell wall hydrolysis. Visualizing enzymes without labeling them and following the evolution of the complex biopolymer network by time-lapse microfluidic FT-IR reflects the actual degradation behavior of the enzymes and the cell walls. The method opens the way to compare enzymes with specific activities, different lignocellulosic biomass sources, and the pretreatments envisioned to overcome degradation recalcitrance. In addition to innovative imaging, the quantification by image analysis and chemometry allows a thorough comparison of different cell types.

## Author contributions

M-FD and FG: planned and designed the research; FJ: designed and supervised the synchrotron fluorescence experiments; WA: designed the microfluidic experiments and supervised the synchrotron infrared microspectroscopy; EB: designed the enzymatic degradation experiments; PR and SD: designed the infrared microspectroscopy experiments; BB, SD, and CA: prepared the samples; BB, CA, SD, EB, M-FD, FG, and WA: performed the experiments; M-FD: wrote the computer code; M-FD, SD, FG, and CA: analyzed the data; FG, EB, and LS: interpreted the hydrolysis behavior; M-FD, FG, SD, and FJ: wrote the manuscript.

### Conflict of interest statement

The authors declare that the research was conducted in the absence of any commercial or financial relationships that could be construed as a potential conflict of interest. The reviewer CY and handling Editor declared their shared affiliation.

## Footnotes

^1^Available online at: https://fr.mathworks.com/products/new_products/release2015a.html

## Supplementary material

Supplementary Data S1 Selection of specific regions for fluorescence intensity measures.

Supplementary Data S4 Difference spectra between Time-lapse FT-IR spectra measured in the microfluidic cell at the beginning of the reaction and after: 15 min for parenchyma below the rind (pr1) and 30 min for parenchyma below the rind (pr2), parenchyma near vascular bundles (pnv), phloem (phl), and xylem parenchyma (pxy) in vascular bundles. All experimental repetitions are represented for each cell type. Y axis scales were adapted for visualization.
