# Supplementary figures and images for: Synchrotron Time-Lapse Imaging of Lignocellulosic Biomass Hydrolysis: Tracking Enzyme Localization by Protein Autofluorescence and Biochemical Modification of Cell Walls by Microfluidic Infrared Microspectroscopy
Source: Front Plant Sci. 2018 Feb 20;9:200. doi: 10.3389/fpls.2018.00200 (PMC5826215; doi:10.3389/fpls.2018.00200)

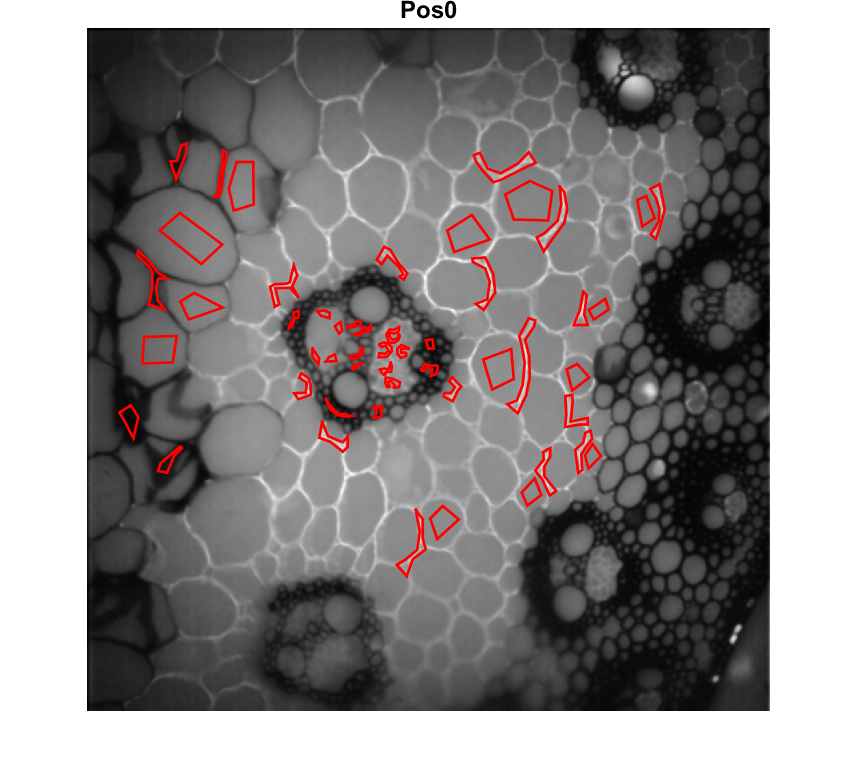

Supplement: Supplementary Data S1 — Selection of specific regions for fluorescence intensity measures. [file SupplementaryDataS1.PNG]

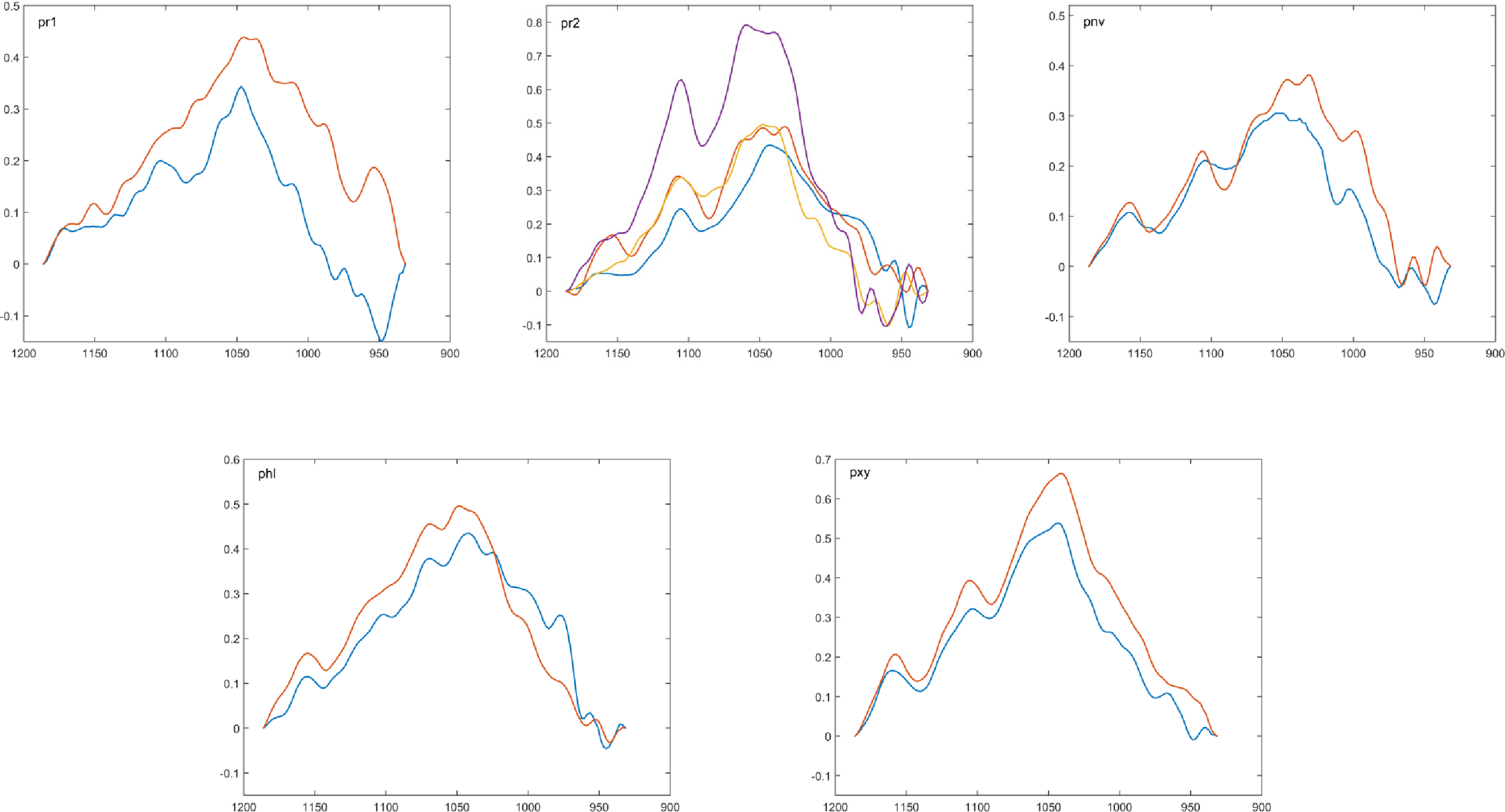

Supplement: Supplementary Data S4 — Difference spectra between Time-lapse FT-IR spectra measured in the microfluidic cell at the beginning of the reaction and after: 15 min for parenchyma below the rind (pr1) and 30 min for parenchyma below the rind (pr2), parenchyma near vascular bundles (pnv), phloem (phl), and xylem parenchyma (pxy) in vascular bundles. All experimental repetitions are represented for each cell type. Y axis scales were adapted for visualization. [file SupplementaryDataS4.TIF]
